# Prevalence and correlates of poor sleep quality among college students: a cross-sectional survey

**DOI:** 10.1186/s12955-020-01465-2

**Published:** 2020-07-01

**Authors:** Yuanyuan Li, Wei Bai, Bo Zhu, Ruixin Duan, Xiao Yu, Wen Xu, Mohan Wang, Wanqing Hua, Weiying Yu, Wenjun Li, Changgui Kou

**Affiliations:** 1 Department of Epidemiology and Biostatistics, School of Public Health, Jilin University, 1163 Xinmin Street, Changchun, 130021 Jilin province China; 2 Department of Social Medicine and Health Management, School of Public Health, Jilin University, Changchun, 130021 China

**Keywords:** Sleep quality, Adolescent, Adult, China

## Abstract

**Background:**

Sleep problems are widespread among college students around the globe, especially in China. This study was designed to investigate the prevalence of poor sleep quality and identify associated factors among college students in Jilin Province, China.

**Methods:**

A total of 6284 participants were completely collected by stratified cluster sampling in 2016. Information on basic demographics, lifestyles, social and family support, and subjective sleep quality was collected by questionnaire. The Pittsburgh Sleep Quality Index (PSQI) is a self-administered questionnaire used to assess sleep for one month.

**Results:**

1951 (31.0%) participants were classified into poor sleep quality group, as defined by a PSQI score > 5. Males scored significantly higher than females on sleep duration and use of sleep medication, while females scored significantly higher than males on PSQI total and sleep disturbances. The results of the multivariate logistic regression show the following factors to be significant predictors of poor sleep quality: freshman (OR = 1.523, 95% CI: 1.168–1.987), alcohol use (OR = 1.634, 1.425–1.874), gambling behaviors (OR = 1.167, 95% CI: 1.005–1.356), exercised for more than 30 min a week on less than one day (OR = 1.234, 95% CI: 1.016–1.498), the feelings of satisfied with parental love (OR = 1.849, 95% CI: 1.244–2.749), and harmonious/neutral relationship with classmates (OR = 2.206, 95% CI: 1.312–3.708; OR = 1.700, 95% CI: 1.414–2.045),. No study pressure of this academic year (OR = 0.210, 95% CI: 0.159–0.276), no truancy in the past month (OR = 0.510, 95% CI: 0.354–0.735), never had self-injurious behaviors (OR = 0.413, 95% CI: 0.245–0.698), very harmonious family relationship (OR = 0.377, 95% CI: 0.219–0.650), frequent communication with parents (OR = 0.524, 95% CI: 0.312–0.880), the feelings of satisfied with maternal love (OR = 0.432, 95% CI: 0.257–0.725), and frequent excursions to gymnasium (OR = 0.770, 95% CI: 0.659–0.899) were the protective factors.

**Conclusions:**

The implication of the present study may be that college students must be made aware of the consequences of inadequate sleep quality and risk factors could be improved if students tried to change their behavior and subjective consciousness.

## Background

Sleep is a pivotal modulator of neuroendocrine function, glucose regulation, and cardiovascular activity. The consequences of sufficient restorative sleep deprivation are severe, impacting human health, wellbeing, and functioning [1]. Poor sleep quality may have a negative impact on social, physical, and mental health, as well as the living quality of individuals*.* According to the cut-off (Pittsburgh Sleep Quality Index (PSQI) score > 5), a German community study indicated that the prevalence of poor sleep quality among people aged 18–80 years was 36% [2]. In a cross-sectional study conducted in Hong Kong, China, the prevalence of poor sleep quality among 5001 adults was 39.4% [3].

As is well-known, college students in the transition period from home to college, from adolescence to adulthood are one of the most sleep-deprived age groups [4]. College students usually face numerous challenges, such as greater academic pressures, social obligations, internet distraction, being responsible for themselves, and erratic schedules. Carskadon and Davis [5] found that students entering the college had less sleep time and delayed sleep onset. Sleep problems can trigger negative health outcomes, such as mood disturbance, fatigue, impaired concentration, and poor academic performance. Sleep problems are widespread among college students around the globe, especially in China. Problems of poor sleep quality and lack of sleep are common among college students in Hong Kong [6]. Li et al. included 82,055 Chinese college students for meta-analysis, with an average sleep time of 7.08 h/day. The proportion of students who slept less than 6 h/day and 7 h/day (short sleep) was 8.4 and 43.9%, respectively. The average bedtime is 12:51 a.m. The proportion of students with large sleep latency (time taken to fall asleep) of more than 30 min was 25.5% [7, 8]. Short sleep duration and unhealthy sleep patterns are common among Chinese university students [9]. Previous studies revealed that a considerable prevalence of poor sleep quality among this population ranged from 19.17 to 57.5% depending on the definition and measure used [10, 11].

Poor sleep quality is associated with a number of factors, including demographic characteristics, behavioral and lifestyle factors, physical activity, psychological factors, and chronic diseases. With age, sleep changes, such as shorter sleep time and increased sleep fragmentation [12]. Zhang et al. found that diseases and increased chronic diseases within 2 weeks in middle-aged and elderly patients were the main physiological health-related factors leading to poor sleep. Physical health may be a major determinant of sleep quality [13]. Wang et al. showed that advanced age, smoking, irregular diet, lack of physical exercise, poor mental health, chronic diseases, or multiple diseases were positively correlated with sleep deprivation [14]. Internet addiction is very prevalent in college students, particularly in Asian communities [15], and is closely associated with sleep problems [16]. In addition, poor sleep quality is also associated with stress levels and education levels [17].

Since the differences in social and cultural background between the East and the West cannot be neglected, the findings may not completely reflect the overall sleep characteristics of the Chinese population, including college students. Many studies on the prevalence of poor sleep quality and associated factors of college students were conducted in China, but other factors such as the family and social support have been overlooked, resulting in failing to obtain complete results.

The purpose of this study was to investigate the prevalence of poor sleep among college students in Jilin Province, China. We looked at factors that influence sleep quality, including demographics and lifestyle, as well as family and social support. Understanding these factors may help improve sleep quality, thereby promoting the development of strategies and raising the quality of life.

## Methods

### Participants and sampling

**Fig. 1:**
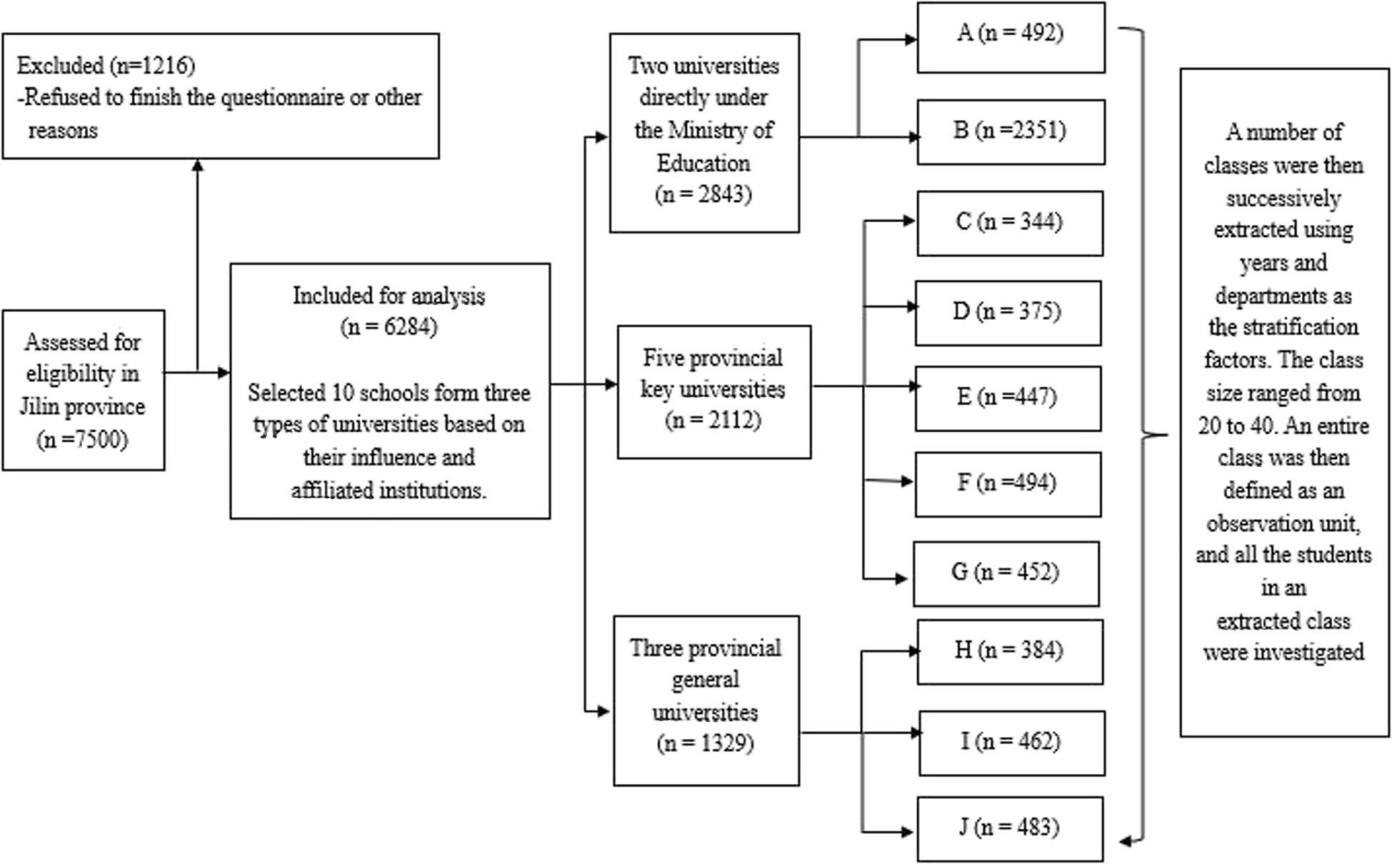
Flow diagram of sampling process

The study was carried out in 2016 in Jilin Province, China. The sample size was 7500 based on the reported prevalence of 30% poor sleep quality, a confidence level of 95%, and an allowable error of 0.0001. Before taking samples, the student roster of different majors and grades was collected, and the students who were absent from the school in the past one month due to their study, sickness and personal leave, and internship were excluded. All the universities included in the survey received permission from the university authorities. Respondents received oral notification of the study from the advisor and were informed that participation was optional. All respondents provided informed consent prior to participating in the study. The survey was conducted anonymously and no personal information was given. 7500 people from the selected sites were randomly selected and invited to participate in the study, accounting for about 1% of the total number of college students in Jilin province. The overall response rate as 95.8% (313 participants refused to respond). The sampling of the survey was conducted by Jilin University using a stratified cluster sampling method. The detailed process of the sampling process was shown in Fig. 1. After the questionnaires were collected, 903 unqualified questionnaires were deleted, 6284 questionnaires included in the final analysis. Multiple imputation was used to deal with missing data for the PSQI scale. Frequency interpolation was used to deal with missing data for ranked data. Mean interpolation and mode interpolation were used to process the missing data in quantitative data of central tendency and discrete tendency, respectively. The study protocol was approved by the Survey and Behavioral Ethics Committee of the School of Public Health, Jilin University.

### Data and collection

PSQI is a self-administered questionnaire used to assess sleep for one month. The index consists of 19 items grouped into 7 components (subjective sleep quality, sleep latency, sleep duration, sleep efficiency, sleep disturbance, daytime dysfunction, and frequency of sleep medications) with 0 (no difficulty) to 3 (severe difficulty). The sum of these 7 components is a global score (range 0–21). The lower the score, the better the quality of sleep. The total score of PSQI > 5 indicates poor sleep quality with a sensitivity of 89.6% and specificity of 86.5% [18]. The Chinese version of PSQI has been widely used in the evaluation of sleep in other regions of China, with good reliability and validity [19].

Information on basic demographic characteristics, lifestyles, social and family support, and subjective sleep quality was collected using a self-report questionnaire. The basic demographic characteristics included age, gender, ethnicity, grade, body mass index (BMI), monthly family per capita income, and monthly living cost. BMI was calculated using self-reported height and weight as weight in kg divided by height in meters squared [20, 21]. Lifestyle factors included tobacco and alcohol use, study pressure during the school year, days off from school, self-injurious behaviors, suicidal ideation in the past 12 months, gambling, and exercise. Tobacco use (at least one cigarette a day a week), alcohol use (one glass of an alcoholic drink, such as half bottle/can of beer, one glass of Chinese liquor, one glass of wine or rice wine) and gambling were categorized into “yes” or “no”. Study pressure was measured by “How about your study pressure and burden this academic year?”. Days off from school was measured by “In the past 30 days, how many days have you missed classes without asking for leave?”. Self-injurious behaviors were measured by “In the past 12 months, have you intentionally hurt yourself (burning a cigarette, cutting with a razor blade, banging your head against a wall, etc.)?”. Suicidal ideation was measured by “Have you had suicidal ideation in the past 12 months?”. Exercise intensity was identified by asking “how many days per week do you exercise for more than 30 minutes at a time?”. Family support is made up of five questions: “How do you feel about your family relationship?”, “What is your parents’ marital status and relationship?”, “How often do you communicate with your parents?”, “How satisfied you are with your father’s love?”, “How satisfied you are with your mother’s love?”. Social support was measured with four questions, “How do you feel about your relationship with your classmates?”, “How many close friends do you have?”, “Where do you often go with your friends?”, “Do you have a boyfriend/girlfriend?” Social and family support was ascertained by asking students to self-evaluate their relationships and satisfaction with parents, classmates, or friends on a five-point scale from very harmonious/satisfaction to highly disharmonious/dissatisfied.

### Statistical analysis

Statistical analyses were performed by SPSS 24.0 (Statistical Product and Service Solutions Inc., Chicago, IL, USA). Basic socio-demographic characteristics, lifestyles, and social and family support were also compared between good and poor sleep quality using univariate logistic analyses for categorical variables. The gender differences for the seven PSQI components were analyzed via t-test. Multi-collinearity diagnosis was conducted for variables with statistical significance. Variables with variance inflation factor (VIF) <3 were included in the multivariate analysis. If there is a collinearity problem, principal component analysis and factor analysis are used to extract common factors and carry out multivariate logistic regression analysis of pre-selected variables. The multivariate logistic regression was used to obtain odds ratios (OR) and 95% confidence intervals (CI) of predictors, and explore the association between factors and sleep quality. Wald test was used to test the hypothesis of the regression coefficient. Two-tailed *p* < 0.05 was applied to determine the statistical significance.

## Results

A total of 6284 eligible questionnaires from undergraduate university student participants were completed in the study. The sample included 52.7% male and 47.3% female. The age of the students ranged from 15 to 25 years, and the mean age was 19.76 years (*SD* = 1.45). The number of students ≤20 years old and > 20 years old was 4550 and 1734 respectively. There were 2586 freshmen, 1966 sophomores, 1072 juniors, and 660 senior and senior five.

Table 1 shows the basic demographic differences between students with poor and good sleep quality. There were 4233 (69.0%) and 1951 (31.0%) with good and poor sleep quality, respectively. The difference in age groups and BMI was non-significant between subjects with good and poor sleep quality (. Students in lower grades had a higher prevalence of poor sleep quality than students in higher grades (*p*<0.05).

**Table 1:** Basic demographic characteristics and sleep quality

Variables	Total (*n* = 6284)	Cr	Good sleep quality (*n* = 4233)	Poor sleep quality (*n* = 1951)	OR	95%CI	*p*
**Age**							
≤ 20	4550	72.4%	3106(68.3)	1444(31.7)	1.000		
> 20	1734	27.6%	1227(70.8)	507(29.2)	1.044	0.871–1.252	0.638
**Gender**							
Male	3310	52.7%	2284(69.0)	1026(31.0)	1.000		
Female	2974	47.3%	2049(68.9)	925(31.1)	1.017	0.888–1.166	0.804
**Ethnicity**							
Han	5737	91.3%	3955(68.9)	1782(31.1)	1.000		
Minority	547	8.7%	378(69.1)	169(30.9)	0.999	0.815–1.225	0.993
**BMI (kg/m**^**2**^**)**							
< 18.5	1158	18.5%	813(70.2)	345(29.8)	1.000		0.228
18.5–23.9	4054	64.5%	2785(68.7)	1269(31.3)	0.769	0.588–1.005	0.055
24–27.9	774	12.3%	543(70.2)	231(29.8)	0.825	0.645–1.056	0.127
≥ 28	298	4.7%	192(64.4)	106(35.6)	0.771	0.581–1.022	0.071
**Grade**							
Freshman	2586	41.2%	1755(67.9)	831(32.1)	1.000		0.002
Sophomore	1966	31.3%	1337(68.0)	629(32.0)	0.873	0.760–1.002	0.054
Junior	1072	17.0%	759(70.8)	313(29.2)	0.677	0.550–0.834	< 0.001
Senior and senior five	660	10.5%	482(73.0)	178(27.0)	0.663	0.508–0.866	0.003
**Monthly family income per capita (¥)**							
< 3000	2909	46.3%	1974(67.9)	1974(32.1)	1.000		0.901
3000–4999	1916	30.5%	1343(70.1)	1343(29.9)	1.029	0.891–1.188	0.695
5000–6999	842	13.4%	589(70.0)	589(30.0)	1.005	0.830–1.217	0.961
≥ 7000	617	9.8%	427(69.2)	427(30.8)	1.085	0.864–1.362	0.481
**Monthly living expenses (¥)**							
< 1000	2036	32.4%	1367(67.1)	669(32.9)	1.000		0.087
1000–2000	3646	58.0%	2537(69.6)	1109(30.4)	0.874	0.762–1.004	0.057
2001–3000	467	7.4%	338(72.4)	129(27.6)	0.829	0.636–1.082	0.168
> 3000	135	2.1%	91(67.4)	44(32.6)	1.245	0.796–1.947	0.336

*Note*: Cr, Constituent ratio; ¥1000 = $141

Table 2 provides for differences in lifestyle factors and subgroups with good or poor sleep quality. Among students who drank alcohol (p<0.001), had higher academic pressure (p<0.001), more days of school absenteeism (p<0.001), had often self-injurious behaviors (p<0.05), gambled (p<0.05), and regularly exercised less than one day a week (p<0.05), there was a higher prevalence of poor sleep quality.

**Table 2:** Univariate logistic regression analysis of lifestyle factors with sleep quality

Variables	Total	Cr	Good sleep quality n(%)	Poor sleep quality n(%)	OR	95%CI	*p*
**Tobacco use**							
yes	354	5.6%	232(65.5)	122(34.5)	1.000		
no	5930	94.4%	4101(69.2)	1829(30.8)	0.927	0.716–1.200	0.564
**Alcohol use**							
yes	4394	69.9%	2880(65.5)	1514(34.5)	1.000		
no	1890	30.1%	1453(76.9)	437(23.1)	0.607	0.528–0.697	< 0.001
**Study pressure of this academic year**							
no	887	14.1%	733(82.6)	154(17.4)	1.000		< 0.001
smaller	903	14.4%	676(74.9)	227(25.1)	1.381	1.082–1.762	0.009
general	2611	41.5%	1894(72.5)	717(27.5)	1.535	1.245–1.893	< 0.001
larger	1488	23.7%	846(56.9)	642(43.1)	3.032	2.437–3.772	< 0.001
great	395	6.3%	184(46.6)	211(53.4)	4.848	3.672–6.401	< 0.001
**Days off from school(/month)**							< 0.001
0	5049	80.3%	3591(71.1)	1458(28.9)	1.000		< 0.001
< 1	574	9.1%	348(60.6)	226(39.4)	1.587	1.307–1.926	< 0.001
1–2	395	6.3%	260(65.8)	135(34.2)	1.371	1.067–1.762	0.014
3–4	110	1.8%	57(51.8)	53(48.2)	2.076	1.366–3.157	< 0.001
≥ 5	156	2.5%	77(49.4)	79(50.6)	1.914	1.326–2.764	< 0.001
**Self-injurious behaviors**							
never	5929	94.4%	4134(69.7)	1795(30.3)	1.000		0.002
Occasionally	196	3.1%	106(54.1)	90(45.9)	1.575	1.141–2.175	0.006
sometimes	75	1.2%	52(69.3)	23(30.7)	0.798	0.444–1.435	0.452
often	84	1.3%	41(48.8)	43(51.2)	2.018	1.165–3.495	0.012
**Suicidal ideation in the past 12 months**							
yes	576	9.20%	338(58.7)	238(41.3)	1.219	0.989–1.503	0.064
no	5708	90.80%	3995(70.0)	1713(30.0)	1.000		
**Gambling behaviors**							
yes	1137	18.1%	734(64.6)	403(35.4)	1.000		
no	5147	81.9%	3599(69.9)	1548(30.1)	0.852	0.732–0.990	0.037
**Exercise for more than 30 min (days/week)**							
0	1526	24.3%	961(63)	565(37)	1.000		0.002
1–2	2687	42.8%	1893(70.5)	794(29.5)	0.768	0.663–0.888	< 0.001
3–4	1128	18.0%	807(71.5)	321(28.5)	0.737	0.612–0.886	0.001
5–7	943	15.0%	672(71.3)	271(28.7)	0.822	0.676–0.999	0.049

*Note*: Cr, Constituent ratio; *P* is for OR

Table 3 shows the family and social support and subgroups with good or poor sleep quality and reveals that students who lacked communication with parents (p<0.05), were dissatisfied with their parental love (p<0.05), and had disharmonious family relationships (p<0.05), were significantly more likely to be poor sleepers. As for social support related factors, often go to Bar/Karaoke hall/Song and dance hall with friends (p<0.05) were also significantly associated with poor sleep quality.

**Table 3:** Univariate logistic regression analysis of family and social support with sleep quality

Variables	Total		Good sleep quality n(%)	Poor sleep quality n(%)	OR	95%CI	*p*
**Family relationship**							
Very harmonious	3884	61.8%	2870(73.9)	1014(26.1)	1.000		< 0.001
Harmonious	1822	29.0%	1142(62.7)	680(37.3)	1.260	1.088–1.459	0.002
Neutral	442	7.0%	262(59.3)	180(40.7)	1.270	0.980–1.647	0.071
Disharmonious	69	1.1%	31(44.9)	38(55.1)	2.026	1.166–3.522	0.012
Highly disharmonious	67	1.1%	28(41.8)	39(58.2)	2.395	1.356–4.228	0.003
**Parental marital status**							
Harmonious	5402	86.0%	3805(70.4)	1597(29.6)	1.000		0.847
Frequent quarrel	494	7.9%	295(59.7)	199(40.3)	0.984	0.778–1.244	0.893
Separation	79	1.3%	46(58.2)	33(41.8)	1.204	0.724–2.001	0.475
Divorce	309	4.9%	187(60.5)	122(39.5)	1.073	0.814–1.415	0.617
**Communication with parents**							
Substantial	1567	24.9%	1186(75.7)	381(24.3)	1.000		< 0.001
Often	2290	36.4%	1594(69.6)	696(30.4)	1.777	1.025–3.079	0.040
Neutral	1857	29.6%	1162(62.6)	695(37.4)	2.511	1.486–4.242	< 0.001
Rarely	398	6.3%	253(63.6)	145(36.4)	2.172	1.285–3.671	0.004
Never	172	2.7%	138(80.2)	34(19.8)	1.997	1.179–3.383	0.010
**Satisfaction with paternal love**							
Very satisfied	3091	49.2%	2252(72.9)	839(27.1)	1.000		0.026
Satisfied	2019	32.1%	1362(67.5)	657(32.5)	1.288	0.756–2.197	0.352
Neutral	786	12.5%	471(59.9)	315(40.1)	0.795	0.500–1.265	0.333
Dissatisfied	174	2.8%	94(54.0)	80(46.0)	0.687	0.437–1.081	0.104
Very dissatisfied	214	3.4%	154(72.0)	60(28.0)	0.702	0.446–1.103	0.125
**Satisfaction with maternal love**							
Very satisfied	3750	59.7%	2677(71.4)	1073(28.6)	1.000		0.011
Satisfied	1855	29.5%	1215(65.5)	640(34.5)	0.698	0.352–1.384	0.304
Neutral	397	6.3%	236(59.4)	161(40.6)	1.553	0.868–2.780	0.138
Dissatisfied	113	1.8%	77(68.1)	36(31.9)	1.688	0.976–2.919	0.061
Very dissatisfied	169	2.7%	128(75.7)	41(24.3)	1.652	0.957–2.852	0.072
**Relationship with classmates**							
Very harmonious	1791	28.5%	1371(76.5)	420(23.5)	1.000		< 0.001
Harmonious	3164	50.4%	2191(69.2)	973(30.8)	1.696	0.762–3.772	0.196
Neutral	1157	18.4%	659(57.0)	498(43.0)	1.308	0.664–2.578	0.438
Disharmonious	80	1.3%	45(56.3)	35(43.8)	0.878	0.446–1.732	0.708
Highly disharmonious	92	1.5%	67(72.8)	25(27.2)	0.785	0.398–1.550	0.486
**Number of good friends**							
None	147	2.3%	91(61.9)	56(38.1)	1.000		0.415
One	233	3.7%	150(64.4)	83(35.6)	0.694	0.413–1.168	0.169
Two	826	13.1%	524(63.4)	302(36.6)	0.793	0.496–1.268	0.332
Three and above	5078	80.8%	3568(70.3)	1510(29.7)	0.730	0.464–1.149	0.174
**Places often going with friends**							
Gymnasium	1662	26.40%	1258(75.7)	404(24.3)	1.000		0.002
Bar/Karaoke hall/Song and dance hall	809	12.90%	509(62.9)	300(37.1)	1.391	1.137–1.702	0.001
Billiard hall	301	4.80%	216(71.8)	85(28.2)	0.996	0.740–1.341	0.979
Internet cafes	630	10.00%	449(71.3)	181(28.7)	1.020	0.814–1.278	0.861
Other	2882	45.90%	1901(66.0)	981(34.0)	1.286	1.101–1.503	0.002
**boyfriend or girlfriend**							
Yes	1587	25.3%	1125(70.9)	462(29.1)	1.000		
No	4697	74.7%	3208(68.3)	1489(31.7)	1.142	0.997–1.308	0.056

*Note*: Cr, Constituent ratio; *P* is for OR

Collinearity diagnosis was conducted for variables with a statistically significant difference in univariate logistic regression analysis, and variables with VIF<3 were included in the multivariable logistic regression (Table 4).

**Table 4:** The diagnosis of multicollinearity in univariate logistic regression analysis of sleep quality of college students

Variables	VIF	Variables	VIF
Gender	1.056	Family relationship	1.288
Alcohol use	1.062	Communication with parents	1.547
Study pressure of this academic year	1.032	Satisfaction with paternal love	2.296
Days off from school(/month)	1.210	Satisfaction with maternal love	2.311
Self-injurious behaviors	1.242	Relationship with classmates	1.240
Gambling behaviors	1.044	Places often going with friends	1.065
Exercise for more than 30 min (days/week)	1.081		

The results of the multivariable logistic regression show that students in the lower grades had an increased risk of poor sleep quality (*p*<0.05). Specifically, freshman and sophomore had a higher risk compared with that senior and senior five (OR = 1.523, 95% CI: 1.168–1.987; OR = 1.327, 95% CI; 1.030–1.709). Alcohol use (OR = 1.634, 1.425–1.874) was significantly associated with poor sleep quality (*p*<0.05). Gambling behaviors (OR = 1.167, 95% CI: 1.005–1.356) was also shown to be a risk factor (*p*<0.05). Students who exercised for more than 30 min a week on less than one day (OR = 1.234, 95% CI: 1.016–1.498) had a higher risk of poor sleep quality than those who exercised for 5 to 7 days a week). Feelings of satisfied with parental (OR = 1.849, 95% CI: 1.244–2.749), and harmonious/neutral relationship with classmates (OR = 2.206, 95% CI: 1.312–3.708; OR = 1.700, 95% CI: 1.414–2.045) were also risk factors (*p*<0.05). Students with study pressure of this academic year had an increased risk during poor sleep quality (*p*<0.001). Students with no study pressure (OR = 0.210, 95% CI: 0.159–0.276) and had the lowest sleep risk than those with great study pressure. Students who did not skip school (OR = 0.510, 95% CI: 0.354–0.735) had a lower risk of poor sleep quality than those who stayed away from school for more than 5 days in the past month. A lower risk was also found for students who never (OR = 0.413, 95% CI: 0.245–0.698) and sometimes (OR = 0.372, 95% CI: 0.180–0.769) had self-injurious behaviors compared to students who often self-injurious behaviors.

In comparison with highly disharmonious family relationships, very harmonious (OR = 0.377, 95% CI: 0.219–0.650), harmonious (OR = 0.473, 95% CI: 0.274–0.817) and neutral family relationships (OR = 0.498, 95% CI: 0.282–0.879) had a lower risk of poor sleep quality. Frequent communication with parents (OR = 0.524, 95% CI: 0.312–0.880), the feelings of satisfied with maternal love (OR = 0.432, 95% CI: 0.257–0.725) and often went to the gymnasium (OR = 0.770, 95% CI: 0.659–0.899) were the protective factors of poor sleep quality (Table 5).

**Table 5:** Multivariable logistic regression of factors associated with poor sleep quality

	Estimate	SE	Wald	*P*	OR	95%CI
**Age**						
≤ 20	−0.043	0.092	0.219	0.640	0.958	0.800–1.147
> 20					1.000	
**Gender**						
Male	0.004	0.067	0.004	0.948	1.004	0.880–1.146
Female					1.000	
**Grade**						
Freshman	0.421	0.136	9.645	0.002	1.523	1.168–1.987
Sophomore	0.283	0.129	4.798	0.028	1.327	1.030–1.709
Junior	0.029	0.123	0.055	0.815	1.029	0.809–1.308
Senior and senior five					1.000	
**Alcohol use**						
yes	0.491	0.070	49.346	< 0.001	1.634	1.425–1.874
no					1.000	
**Study pressure of this academic year**						
no	−1.563	0.140	123.709	< 0.001	0.210	0.159–0.276
smaller	−1.249	0.134	87.531	< 0.001	0.287	0.221–0.373
general	−1.150	0.117	96.568	< 0.001	0.317	0.252–0.398
larger	−0.464	0.120	14.857	< 0.001	0.629	0.497–0.796
great					1.000	
**Days off from school(/month)**						
0	−0.673	0.186	13.091	< 0.001	0.510	0.354–0.735
< 1	−0.214	0.204	1.102	0.294	0.807	0.541–1.204
1–2	−0.381	0.213	3.189	0.074	0.683	0.450–1.038
3–4	0.044	0.269	0.027	0.870	1.045	0.617–1.770
≥ 5					1.000	
**Self-injurious behaviors**						
never	−0.883	0.268	10.897	0.001	0.413	0.245–0.698
Occasionally	−0.389	0.302	1.666	0.197	0.678	0.375–1.224
sometimes	−0.988	0.370	7.123	0.008	0.372	0.180–0.769
often					1.000	
**Gambling behaviors**						
yes	0.154	0.076	4.082	0.043	1.167	1.005–1.356
no					1.000	
**Exercise for more than 30 min (days/week)**						
0	0.210	0.099	4.512	0.034	1.234	1.016–1.498
1–2	−0.067	0.090	0.551	0.458	0.935	0.784–1.116
3–4	−0.107	0.104	1.048	0.306	0.899	0.732–1.103
5–7					1.000	
**Family relationship**						
Very harmonious	−0.975	0.278	12.292	< 0.001	0.377	0.219–0.650
Harmonious	−0.748	0.278	7.225	0.007	0.473	0.274–0.817
Neutral	−0.698	0.290	5.783	0.016	0.498	0.282–0.879
Disharmonious	−0.191	0.372	0.264	0.608	0.826	0.398–1.714
Highly disharmonious					1.000	
**Communication with parents**						
Substantial	−0.646	0.265	5.969	0.015	0.524	0.312–0.880
Often	−0.106	0.148	0.513	0.474	0.899	0.672–1.203
Neutral	0.223	0.096	5.371	0.020	1.249	1.035–1.508
Rarely	0.077	0.084	0.827	0.363	1.080	0.915–1.274
Never					1.000	
**Satisfaction with paternal love**						
Very satisfied	0.360	0.228	2.487	0.115	1.433	0.916–2.240
Satisfied	0.615	0.202	9.227	0.002	1.849	1.244–2.749
Neutral	0.134	0.120	1.253	0.263	1.144	0.904–1.448
Dissatisfied	−0.020	0.092	0.049	0.825	0.980	0.818–1.174
Very dissatisfied					1.000	
**Satisfaction with maternal love**						
Very satisfied	−0.499	0.278	3.238	0.072	0.607	0.352–1.046
Satisfied	−0.840	0.265	10.074	0.002	0.432	0.257–0.725
Neutral	−0.045	0.151	0.090	0.765	0.956	0.712–1.284
Dissatisfied	0.016	0.091	0.030	0.863	1.016	0.850–1.214
Very dissatisfied					1.000	
**Relationship with classmates**						
Very harmonious	0.412	0.317	1.686	0.194	1.509	0.811–2.809
Harmonious	0.791	0.265	8.912	0.003	2.206	1.312–3.708
Neutral	0.531	0.094	31.805	< 0.001	1.700	1.414–2.045
Disharmonious	0.107	0.076	1.972	0.160	1.113	0.958–1.293
Highly disharmonious					1.000	
**Places often going with friends**						
Gymnasium	−0.261	0.079	10.914	0.001	0.770	0.659–0.899
Bar/Karaoke hall/Song and dance hall	0.080	0.092	0.768	0.381	1.084	0.905–1.297
Billiard hall	−0.258	0.148	3.056	0.080	0.773	0.579–1.032
Internet cafes	−0.259	0.110	5.502	0.019	0.772	0.622–0.958
Other					1.000	

*Note: df* = 1

Differences between the genders for the seven PSQI components are depicted in Appendix 2. The mean sleep quality score was 4.51 (*SD* = 2.52), the median sleep latency was 10.0 min (P25-P75, 5.0–20.0), the mean sleep efficiency was 96.21% (*SD* = 3.85), the mean sleep duration was 7.47 h (*SD* = 1.15), and of them, 1.97, 9.01, 29.46, and 59.56% were in < 5/5−/6−/> 7(h) sleep duration subgroups, respectively. Table 5 shows that males scored significantly higher than females on sleep duration (*p =* 0.012) and use of sleep medication (*p =* 0.013), while females scored significantly higher than males on PSQI total (*p =* 0.041) and sleep disturbances (*p* < 0.001). No significant difference was observed in subjective sleep quality, sleep latency, sleep efficiency, and daytime dysfunction between males and females.

## Discussion

The present study explored the prevalence and associated factors of poor sleep quality among college students in Jilin province, China. With the cut-off (PSQI > 5), our findings of the PSQI total mean score of 4.51 (*SD* = 2.52) and the 31.0% prevalence of poor sleep quality were similar to those found in a study conducted in Taiwan university students [22], who had the PSQI total mean score of 4.9 (*SD* = 2.4) and a 33.8% prevalence of poor sleep quality. Furthermore, our result is lower than the prevalence of poor sleep quality in a general university sample in Ethiopia (55.8%) [23] and in Hong Kong (57.5%) [10], but higher than another study of medical students in China (19.2%) [11].

Based on the basic demographic results, students in lower grades had a higher prevalence of poor sleep quality compared with those in the higher grades. Freshmen might be more susceptible to the new freedoms of living away from home for the first time and have little experience in dealing with the academic rigor of the curriculum. Similar results were found in the previous study [24]. No significant differences were found between males and females in the prevalence of poor sleep quality; this seems to contradict other previous reports [3, 25]. We also found no association between sleep quality and BMI in college students, while other studies reported sleep quality had a significant association with BMI in the general population [26, 27]. Ethnicity, age structure, region, socioeconomic level, and lifestyle might be partly responsible for the differences [13, 26, 28, 29].

Regarding lifestyle, in the present study poor sleep quality was found to be associated with alcohol use, study pressure, days off from school, self-injurious behaviors, suicidal ideation, gambling, and physical exercise. Smoking is considered as a negative factor for improving sleep quality [30], but this was not observed in our study.

This work also found that poor sleep quality was associated with family and social support. A study demonstrated that family and social support may be an important determinant of sleep quality in the elderly [30, 31]. Daytime emotional stress, which may be disrupted by the interpersonal environment, has a strong relationship with sleep quality [32]. Therefore, this study found the association between social support and sleep quality is reasonable.

Our multiple logistic regression results also showed that lower grade, disharmonious family relationships, less frequent communication with parents, alcohol use, study pressure, more days of school absenteeism, self-injurious, suicidal ideation, and physical exercise more often could increase the odds of poor sleep quality after controlling gender and age.

The sleep quality of female students was not worse than that of male students in terms of global PSQI scores and sleep disturbances. The only two significant gender findings were that male students had worse sleep quality in terms of sleep duration and use of sleep medication relative to female students.

Nevertheless, there existed some limitations. First, due to the cross-sectional design of this study, the causal relationship between sleep quality and factors could not be determined; further multi-center and longitudinal studies need to be done. Second, the study only covered college students in just Jilin Province, so the results may not necessarily be generalized to the whole country’s undergraduate population or the Chinese college-aged population. Third, we cannot rule out the possibility that our results are due to unmeasured variables (such as dietary intake, use of the Internet and chronic diseases) that might affect sleep quality, or to chance. Fourth, this study used a self-reported approach to collect data, including height, weight, and sleep quality, which may be subject to some reporting errors. However, the simple self-estimation of the height and weight of college students is accurate enough to be used in place of measurement [21]. Moreover, the validated and structured questionnaire has been regularly incorporated into the large epidemiologic field surveys [33]. Fifth, the methods used to access some of the questions in the questionnaire may not be accurate enough. For example, exercise intensity was measured by “how many days per week do you exercise for more than 30 minutes at a time?”. There may be a lack of evidence that family and social support measures have validity and sound psychometric properties. Finally, the limitations of using PSQI to assess sleep quality studies also have been pointed out by other studies, despite its widespread use. The previous study demonstrated that the PSQI sleep parameters appeared to be more biased compared with the Self-Assessment of Sleep Survey and the Self-Assessment of Sleep Survey Split [34].

## Conclusions

In sum, our results could still serve as an important implication to identify the factors that affect poor sleep and to develop prevention strategies for college students to promote healthy sleeping habits, which should cover factors such as study pressure, family and social support, mental health, and physical exercise. It is worth noting that college students must be made aware of the consequences of inadequate sleep quality and that risk factors could be improved if students tried to change their behavior and subjective consciousness.

## Supplementary information

**Additional file 1 Appendix 1**. The assignment of the variables included in the logistic regression. **Appendix 2**. PSQI component scores and total scores (M ± SD) in all participants and by gender.

## Data Availability

The datasets used and/or analyzed during the current study are available from the corresponding author on reasonable request.

## Abbreviations

PSQI: Pittsburgh Sleep Quality Index
VIF: Variance inflation factor
BMI: Body mass index
OR: Odds ratios
CI: Confidence intervals
SD: Standard deviation
