# Supplementary material for: Prevalence and correlates of poor sleep quality among college students: a cross-sectional survey
Source: Health Qual Life Outcomes. 2020 Jul 1;18:210. doi: 10.1186/s12955-020-01465-2 (PMC7329462; doi:10.1186/s12955-020-01465-2)
Supplement: Supplementary file 1 — Additional file 1 Appendix 1. The assignment of the variables included in the logistic regression. Appendix 2. PSQI component scores and total scores (M ± SD) in all participants and by gender. [file 12955_2020_1465_MOESM1_ESM.docx]

**Appendix 1 The assignment of the variables included in the logistic regression**

| Code | Variable | Assignment |  |  |  |  |
| --- | --- | --- | --- | --- | --- | --- |
| Y | Sleep quality | 0=≤5 | 1=＞5 |  |  |  |
| X1 | Age | 1=≤20 | 2=＞20 |  |  |  |
| X2 | Gender | 1=Male | 1=Female |  |  |  |
| X3 | Ethnicity | 1=Han | 2=Minority |  |  |  |
| X4 | BMI | 0=＜18.5 | 1=18.5-23.9 | 2=24-27.9 | 3=≥28 |  |
| X5 | Grade | 1=Freshman | 2=Sophomore | 3=Junior | 4=Senior and above |  |
| X6 | Monthly family income per capita(￥) | 1=＜3000 | 2=3000-4999 | 3=5000-6999 | 4=≥7000 |  |
| X7 | Monthly living expenses (¥) | 1=＜1000 | 2=1000-2000 | 3=2001-3000 | 4=＞3000 |  |
| X8 | Tobacco use | 1=Yes | 2=No |  |  |  |
| X9 | Alcohol use | 1=Yes | 2=No |  |  |  |
| X10 | Study pressure of this academic year | 1=No | 2=Smaller | 3=General | 4=Larger | 5=Great |
| X11 | Days off from school(/month) | 1=0 | 2=＜1 | 3=1-2 | 4=3-4 | 5=≥5 |
| X12 | Self-injurious behaviors | 1=Never | 2=Occasionally | 3=Sometimes | 4=Often |  |
| X13 | Suicidal ideation in the past 12 months | 1=No | 2=Yes |  |  |  |
| X14 | Gambling behaviors | 1=Yes | 2=No |  |  |  |
| X15 | Exercise for more than 30 minutes(days/week) | 1=0 | 2=1-2 | 3=3-4 | 4=5-7 |  |
| X16 | Family relationship | 1=Very harmonious | 2=Harmonious | 3=Neutral | 4=Disharmonious | 5=Highly disharmonious |
| X17 | Parental marital status | 1=Harmonious | 2=Frequent quarrel | 3=Separation | 4=Divorce |  |
| X18 | Communication with parents | 1= Substantial | 2=Often | 3=Neutral | 4=Rarely | 5=Never |
| X19 | Satisfaction with paternal love | 1=Very satisfied | 2=Satisfied | 3=Neutral | 4=Dissatisfied | 5=Very dissatisfied |
| X20 | Satisfaction with maternal love | 1=Very satisfied | 2=Satisfied | 3=Neutral | 4=Dissatisfied | 5=Very dissatisfied |
| X21 | Relationship with classmates | 1=Very harmonious | 2=Harmonious | 3=Neutral | 4=Disharmonious | 5=Highly disharmonious |
| X22 | Number of good friends | 1=None | 2=One | 3=Two | 4=Three and above |  |
| X23 | Places often going with friends | 1=Gymnasium | 2=Bar/Kara oke hall/Song and dance hall | 3=Billiard hall | 4=Internet cafes | 5=Other |
| X24 | boyfriend or girlfriend | 1=Yes | 2=No |  |  |  |

**Appendix 2** PSQI component scores and total scores (M±SD) in all participants and by gender.

| Variables | Total | male | female | *t* | *p* |
| --- | --- | --- | --- | --- | --- |
| PSQI total | 4.51±2.52 | 4.45±2.52 | 4.58±2.51 | -2.043 | 0.041 |
| Subjective sleep quality | 1.05±0.76 | 1.05±0.77 | 1.04±0.76 | 0.402 | 0.688 |
| Sleep latency | 0.68±0.78 | 0.66±0.78 | 0.69±0.79 | -1.259 | 0.208 |
| Sleep duration | 0.53±0.74 | 0.56±0.75 | 0.51±0.73 | 2.521 | 0.012 |
| Sleep efficiency | 0.02±0.18 | 0.02±0.18 | 0.02±0.17 | -0.060 | 0.952 |
| Sleep disturbances | 0.64±0.57 | 0.57±0.57 | 0.71±0.57 | -9.549 | ＜0.001 |
| Use of sleep medication | 0.04±0.26 | 0.04±0.29 | 0.03±0.22 | 2.491 | 0.013 |
| Daytime dysfunction | 1.56±0.91 | 1.55±0.93 | 1.58±0.89 | -1.675 | 0.094 |
